# Unveiling novel insights into gene expression in monkey testes through single-nucleus-based analysis

**DOI:** 10.3389/fcell.2026.1930189

**Published:** 2026-08-19

**Authors:** Yu Zhang, Shu Wei, Runqing Zou, Yuxin Li, Nianfeng Jiang, Zhao Li, Chao Ma, Yun Wang, Jin Ben, Longbao Lv, Fang Yan

**Affiliations:** 1 School of Life Sciences, Yunnan University, Kunming, China; 2 Yunnan Institute of Pediatrics, Yunnan Key Laboratory of Children’s Major Disease Research, Yunnan Province Clinical Research Center for Children’s Health and Disease, Kunming Children’s Hospital, Kunming, China; 3 Laboratory Animal Center, Kunming Institute of Zoology, Chinese Academy of Sciences, Kunming, China

**Keywords:** sertoli cells, single-nucleus ATAC-seq, single-nucleus RNA-seq, spermatogenesis, spermatogonial stem cells

## Abstract

**Background:**

Spermatogenesis is a highly intricate and tightly regulated process that involves the coordinated interplay of various cell types. Understanding the expression profiles of individual cells is crucial for unraveling the intricacies of spermatogenesis. This study aimed to uncover novel gene expression patterns and regulatory features during primate spermatogenesis.

**Methods:**

We performed single-nucleus RNA sequencing on testicular tissues from cynomolgus macaques at four developmental stages (infancy, puberty, adulthood, and aged). To explore chromatin regulatory landscapes, we also conducted single-nucleus ATAC sequencing on pubertal testes. We integrated both datasets and applied clustering, differential expression analysis, gene ontology enrichment, pseudo time trajectory reconstruction, and transcription factor motif enrichment to characterize cell types, differentiation trajectories, and cell-type-specific regulatory programs.

**Results:**

Our integrated analysis comprehensively characterized all major germ and somatic cell types, including a previously unrecognized quiescent undifferentiated spermatogonial (uSPG) subtype in early spermatogenesis that exhibits a transcriptomic state distinct from canonical type A spermatogonial stem cells, which we provisionally term as uSPG1. We also revealed the differentiation states, and expression and function of genes within distinct cell types during spermatogenesis. Notably, we found that classic niche factors essential for spermatogonial stem cell maintenance, including *GDNF*, *FGF2*, and *CXCL12*, were not readily detected in Sertoli cells by snRNA-seq. Instead, their transcripts were predominantly observed in myoid cells or germ cells, a pattern corroborated by both transcriptomic and chromatin accessibility data. This observation, pending further experimental validation, suggests a need for reevaluation of the cellular sources of these critical niche signals in primates.

**Conclusion:**

These findings offer a refined single-nucleus-resolution view of primate spermatogenesis, revealing a novel undifferentiated germ cell state and emphasizing the need for further exploration of Sertoli cells in male reproductive biology. Our results underscore the power of combined single-nucleus RNA and ATAC sequencing for investigating complex reproductive tissues. This research enhances our understanding of the intricate mechanisms governing spermatogenesis and may offer new perspectives on male fertility regulation.

## 1 Introduction

Spermatogenesis is a highly complex and tightly regulated process essential for male fertility and reproduction. It involves a meticulously orchestrated interplay of various cell types within the testes, each contributing to the intricate process of germ cell development and maturation. Understanding the expression profiles and regulatory mechanisms of these individual cells is essential for unraveling the complexities of spermatogenesis and shedding light on male reproductive biology. Among the various cell types involved in spermatogenesis in testes, germ cells undergo a highly ordered developmental process, progressing from undifferentiated spermatogonia including spermatogonial stem cells (SSCs) and progenitor spermatogonia, to differentiating and differentiated spermatogonia, then entering meiosis as spermatocytes, and finally giving rise to post-meiotic spermatids (Edenfield et al., 2025). Somatic cells provide essential structural and nutritional support for spermatogenesis. Specifically, Sertoli cells play a pivotal role in providing essential factors for germ cell growth and maturation. Sertoli cells not only form the structural framework of the seminiferous tubules but also secrete various growth factors, cytokines, and hormones that regulate germ cell proliferation, differentiation, and survival (Griswold, 2018). The intricate crosstalk between Sertoli cells and germ cells is crucial for the successful progression of spermatogenesis.

Recent advancements in single-cell genomics have provided unprecedented insights into the cellular heterogeneity and regulatory networks governing spermatogenesis. Remarkable progress has been made in the field of testicular single-cell transcriptomics, with notable advancements in mice (Chen et al., 2018; Lin et al., 2026), monkeys (Lau et al., 2020; Shami et al., 2020), and humans (Shami et al., 2020; Guo et al., 2018; Sohni et al., 2019). These studies have yielded profound insights into the intricate dynamics of cellular heterogeneity within the testicular microenvironment. However, conventional single-cell manipulation often results in cell integrity damage during the digestion step, particularly within the seminiferous tubules where Sertoli cells and germ cells are closely interconnected. The promising avenue to address this challenge hinges on the implementation of single-nucleus transcriptomics (Bakken et al., 2018; Slyper et al., 2020). Emerging evidence underscores a remarkable alignment between the datasets derived from single-nucleus and single-cell transcriptomes. This convergence implies that a nuanced comprehension of gene expression dynamics at the single-cell level can be achieved through meticulous single-nucleus transcriptome analysis, circumventing the inherent risks associated with conventional single-cell manipulations that may compromise cellular integrity.

In this study, we aim to unveil novel insights into the intricate nature of spermatogenesis within the testicular microenvironment of non-human primates. Specifically, we utilized single-nucleus transcriptomics to comprehensively investigate the gene expression profiles within different cell types, particularly Sertoli cells and germ cells, across various stages of spermatogenesis. By integrating this approach with single-nucleus ATAC data, we sought to explore the regulatory landscape governing the expression of these cells during the crucial phase of puberty. This research not only advances our fundamental understanding of spermatogenesis but also holds promise for insights into male reproductive health.

## 2 Results

### 2.1 Single-nucleus transcriptomes of monkey testes

**FIGURE 1:**
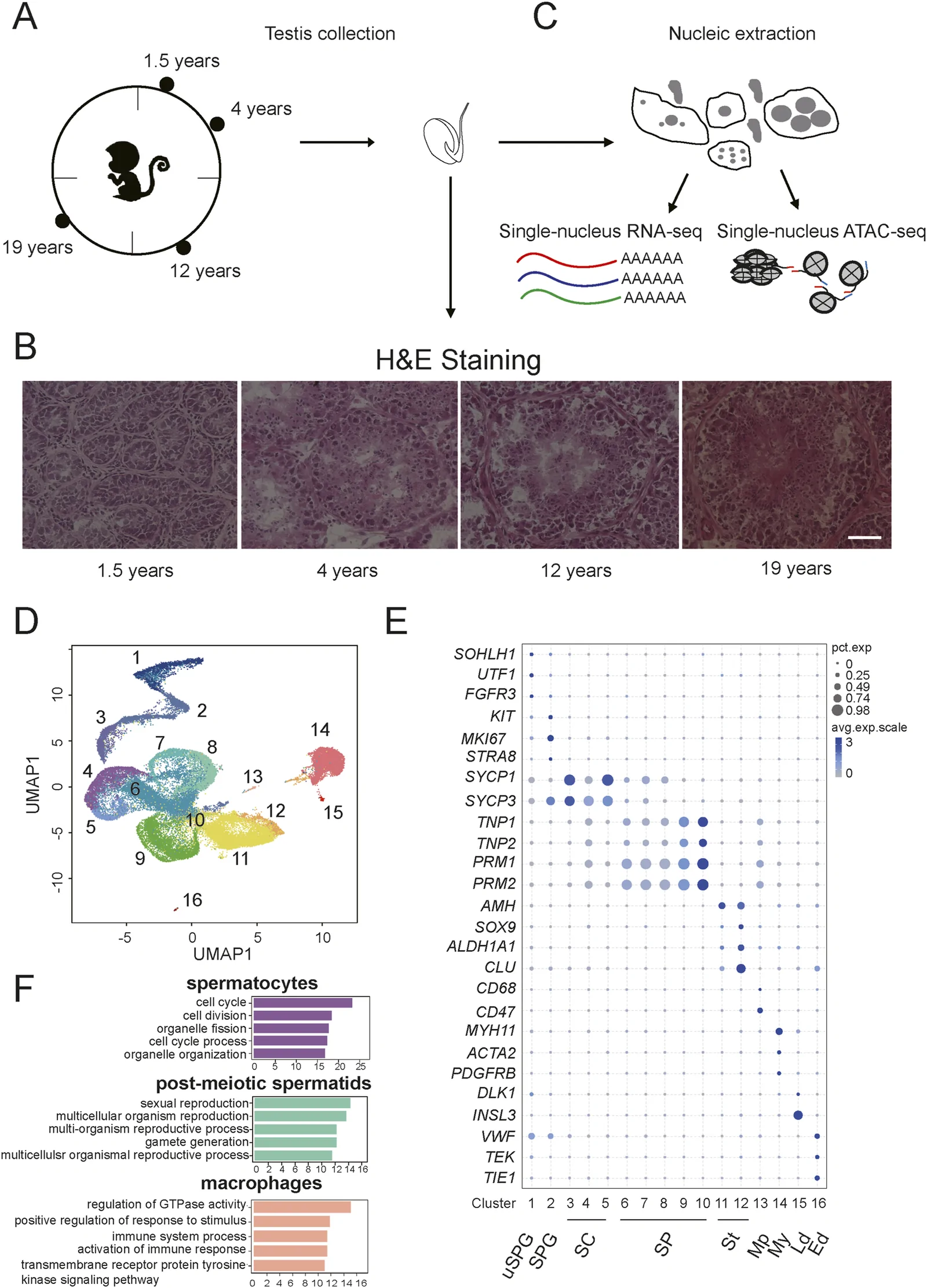
Single-nucleus transcriptomic landscape of monkey testes across different ages. **(A)** Schematic of the experimental design: testicular tissues were collected from cynomolgus macaque at infancy (1.5 years old), puberty (4 years old), adult (12 years old), and aged (19 years old). **(B)** Haematoxylin and eosin staining showing progressive morphological changes of seminiferous tubules with age. Scale bar, 25 µm. **(C)** Workflow of single-nucleus RNA-seq and single-nucleus ATAC-seq analyses. **(D)** UMAP plot of 43,902 high-quality nuclei from all four age groups, colored by 16 major clusters. **(E)** Dot plot showing marker gene expression used to annotate cell types. Abbreviations: uSPG, undifferentiated spermatogonia; SPG, differentiating/differentiated spermatogonia; SC, spermatocytes; SP, spermatids; St, Sertoli cells; Mp, macrophages; My, myoid cells; Ld, Leydig cells; Ed, endothelial cells. **(F)** GO enrichment analysis of marker genes for selected cell types, confirming the functional identities of the clusters.

In order to study the process of spermatogenesis in monkeys, we collected testicular tissues from four different age groups of the cynomolgus macaque: infancy (1.5 years old), puberty (4 years old), adult (12 years old), and aged (19 years old) (Figure 1A). The histological examination revealed major changes in the morphology and composition among these testes (Figure 1B). In infant (1.5 years old) testis, seminiferous tubules lack the apparent lamina or lumen. In pubertal (4 years old) testis, the testis gradually matured. A tubular structure became progressively apparent and began to produce sperm. While a clearly defined lamina and lumen were observed across seminiferous tubules of the adult and the aged (12 and 19 years old) testes. These observations are consistent with previous identifications (Kluin et al., 1983).

To characterize the molecular features across this developmental progression, we prepared single-nucleus suspensions from these testicular tissues and performed single-nucleus RNA sequencing using the 10x Genomics platform (Figure 1C). From a total of 45,834 nuclei, 43,902 passed standard quality control (QC) dataset filters and were retained for downstream analysis. We first performed UMAP (uniform manifold approximation and projection dimension reduction analysis) on the combined datasets from all four age groups using the Seurat package (Butler et al., 2018), which yielded 16 major clusters that were subsequently annotated using known gene markers (Figures 1D,E). Clusters 1–10 are germ cells. Cluster 1 represents undifferentiated spermatogonia (uSPG), which uniquely expresses *SOHLH1*, *UTF1*, and *FGFR3.* Cluster 2 corresponds to differentiating and differentiated spermatogonia (SPG), with expression of *KIT*, *MKI67*, and *STRA8*. Cluster 3–5 consists of spermatocytes (SC), which express *SYCP1* and *SYCP3*. Clusters 6–10 are post-meiotic spermatids (SP), with expression of *TNP1*, *TNP2*, *PRM1*, and *PRM2*. Clusters 11–16 correspond to somatic cells. Clusters 11–12 represent Sertoli cells (St), with the unique expression of *AMH*, *SOX9*, *ALDH1A1*, and *CLU*. Cluster 13 is macrophage (Mp), which expresses *CD68* and *CD47*. Cluster 14 is myoid cell (My), with expression of *MYH11*, *ACTA2*, and *PDGFRB*. Cluster 15 is a small population of Leydig cells (Ld), with the expression of *DLK1* and *INSL3.* Cluster 16 is endothelial cells (Ed), with expression of *VWF*, *TEK*, and *TIE1*.

To annotate gene expression patterns of different cell types based on snRNA-seq, we performed differential expression (DE) testing across these groups. Then the differential expressed genes were subsequently used to perform gene ontology (GO) enrichment analyses. As expected, specifically expressed genes of different cell types could be characterized by responding biological processes (Supplementary Figure S1A). For example, the biological process of male gamete generation and sexual reproduction are enriched in post-meiotic spermatids (Figure 1F). Also, the immune process is enriched in macrophages (Figure 1F). The GO enrichment analysis further confirmed the cell annotation by gene markers.

Subsequently, we proceeded to analyze the expression profiles of representative cell type markers identified in single-cell transcriptomic datasets (Lau et al., 2020; Shami et al., 2020) within the context of single-nuclear transcriptome clustering. Most of the marker genes from scRNA-seq dataset were enriched in the same cell types in our snRNA-seq dataset (Supplementary Figure S1B, S1C). However, a few marker genes identified by Shami et al., 2020, exhibited notably different expression patterns between the scRNA-seq and snRNA-seq, such as *NR6A1*, *MORC1*, and *BRDT* (Supplementary Figure S1C).

### 2.2 Single-cell chromatin accessibility of the pubertal monkey testes

**FIGURE 2:**
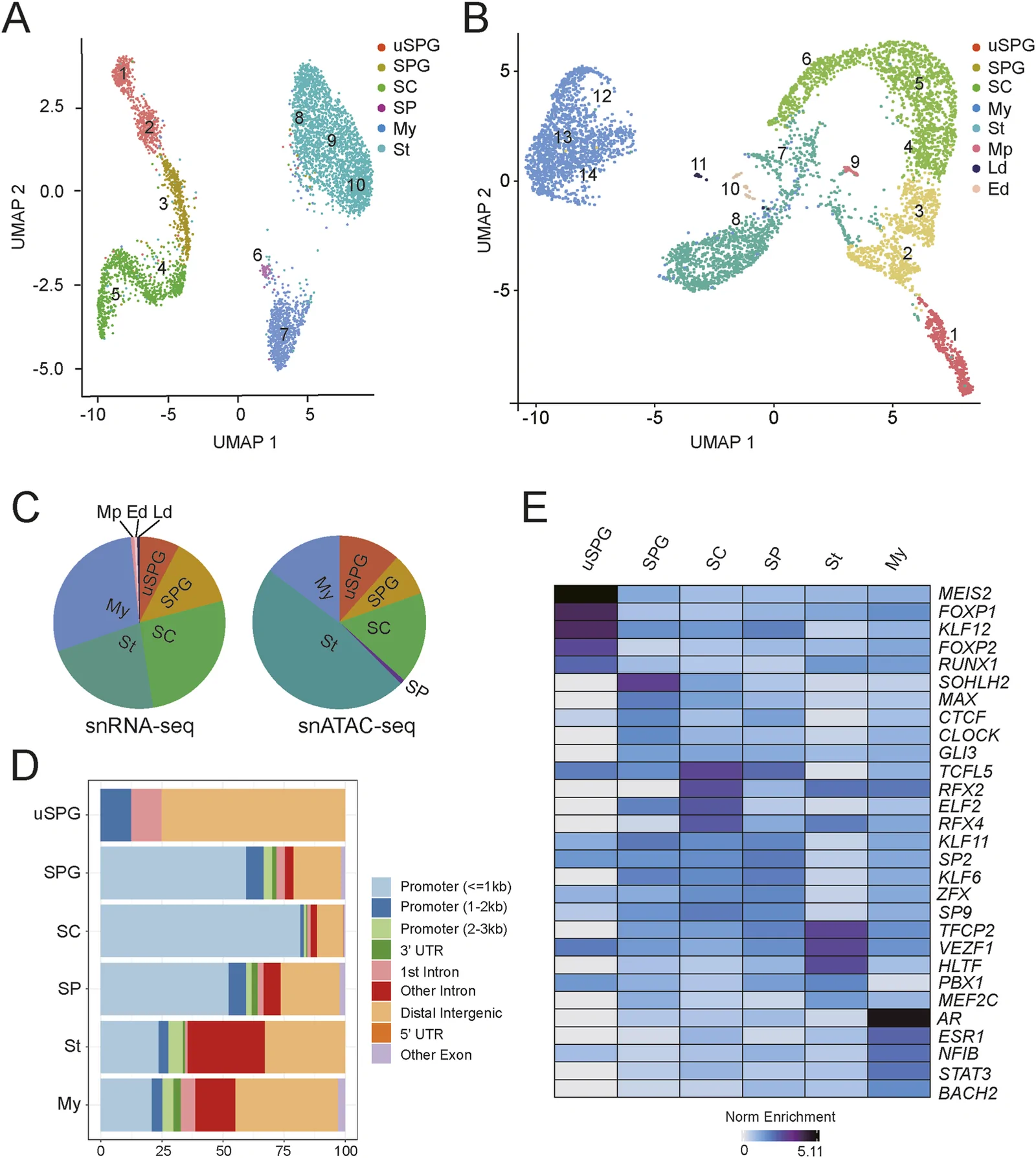
Single-nucleus chromatin accessibility landscape of pubertal monkey testis and integration with snRNA-seq. **(A)** UMAP plot of 5,103 high-quality nuclei from the pubertal (4 years old) testis based on snATAC-seq, colored by cell types. **(B)** UMAP plot of snRNA-seq data from the same pubertal testis for comparison. **(C)** Integration of snRNA-seq and snATAC-seq dataset. Consistent cell-type assignment was identified for uSPG, SPG, SC, St and My. Mp, Ld and Ed were only identified by snRNA-seq, and SP was only identified by snATAC-seq. **(D)** Feature distribution of chromatin-accessible regions across cell types. **(E)** Heatmaps of transcription factor motif enrichment in each cell type identified by snATAC-seq.

To understand the regulatory landscape of individual cells in the testes, we investigated chromatin accessibility for crucial phase of pubertal (4 years old) testis at a single-nucleus level based on single-nucleus ATAC-seq (Figures 1A,C). Single-nucleus profiles consisted of high-quality 5,103 nuclei passing stringent quality filtering. By analyzing the chromatin accessibility profiles of these nuclei, they were grouped into 10 clusters corresponding to 6 cell types (Figure 2A; Supplementary Figure S2A). Four cell types comprised of germ cells: undifferentiated spermatogonia (uSPG) with opened chromatic accessibility of *FGFR3* and *SOHLH1* but closed chromatic accessibility of *KIT*, *MKI67*, and *STRA8*; differentiating and differentiated spermatogonia (SPG) with opened chromatic accessibility of *KIT*, *MKI67*, and *STRA8*; spermatocytes (SC) with opened chromatic accessibility of *SYCP1* and *SYCP3*; post-meiotic spermatids (SP) with opened chromatic accessibility of *TNP2*. Two types of somatic cells were annotated based on snATAC-seq: Sertoli cells (St) with opened chromatic accessibility of *SOX9* and *CLU*; myoid cells (My) with opened chromatic accessibility of *MYH11*, *ACTA2*, and *PDGFRB*.

In order to integrate the analysis of snATAC-seq and snRNA-seq data, we exclusively focused our analyses on snRNA-seq obtained from pubertal (4 years old) testis (Figure 2B, Supplementary Figure S2B). We found that most cell types identifiable by snRNA-seq were also captured based on snATAC-seq including uSPG, SPG, SC, St and My (Figure 2C). Both datasets predominantly captured spermatocytes (SC) as the most abundant germ cells. A small number of post-meiotic spermatids (SP) were annotated using snATAC-seq, while they were not identified in the snRNA-seq data (Figure 2C). Both the snRNA-seq and snATAC-seq captured a considerable proportion of somatic cells, particularly highlighting Sertoli cells and myoid cells. Nevertheless, cell types such as macrophages, Leydig cells, and endothelial cells were exclusively identifiable by snRNA-seq.

Next, we delved into the differences in chromatin accessibility among these distinct cell types. By examining the discrepant peaks of genomic accessible regions, we observed unique patterns of peak distribution across different cell types (Figure 2D). Specifically, for SPG, SC and SP, the majority of regulatory regions influencing gene expression were concentrated within the proximal promoter regions (≤1 kb). In comparison, a notable increased portion of regulatory regions for SP were identified in distal intergenic regions. As for uSPG, the predominant regulatory regions were distributed in distal intergenic regions. Both Sertoli cells (St) and myoid cells (My) predominantly harbored regulatory regions in proximal promoter locations, introns, and distal intergenic regions, with a slightly lower proportion of introns observed in My cells.

Through the integration of motif enrichment with differential expression (DE) analysis, we have identified key transcription factors potentially influencing each cell type (Figure 2E). Notably, *MEIS2*, *FOXP1*, *KLF12*, *FOXP2*, and *RUNX1* were significantly enriched in uSPGs, while *SOHLH2*, *MAX*, *CTCF*, *CLOCK*, and *GLI3* were notably enriched in SPGs. The SCs exhibited significant enrichment of *TCFL5*, *RFX2*, *ELF2*, and *RFX4*, whereas the SPs displayed significant enrichment of *KLF11*, *SP2*, *KLF6*, *ZFX*, and *SP9*. Several of these transcription factors, such as *SOHLH2*, *CTCF, GLI3*, *TCFL5*, *RFX2*, *RFX4*, *SP2*, and *SP9*, have been previously linked to spermatogenesis (Ballow et al., 2006; Cecchini et al., 2023; Hernández-Hernández et al., 2016; Kistler et al., 2015; Persengiev et al., 1997; Wolfe et al., 2008; Xiao et al., 2002). Additionally, *PBX1*, *MEF2C*, *AR*, *ESR1*, and *STAT3*, known to be associated with spermatogenesis, were significantly enriched in expression and motif in somatic cells. Although some of the enriched genes have not been directly linked to spermatogenesis, they may still have a role in this biological process. For example, the *FOXP* family of proteins is known to be involved in various differentiation processes (Gao et al., 2023), whereas the *KLF* family is associated with pluripotency (Bialkowska et al., 2017). Further investigation is necessary to elucidate the specific functions of these transcription factors in spermatogenesis.

### 2.3 Subclustering of germ cells in pubertal monkey testes

In order to enhance our understanding of the transcriptional changes and regulation of germ cells during spermatogenesis, we conducted subclustering analysis on the snRNA-seq and snATAC-seq for germ cells in pubertal monkeys. For consistency, snATAC-seq data was filtered to exclude SP cells.

**FIGURE 3:**
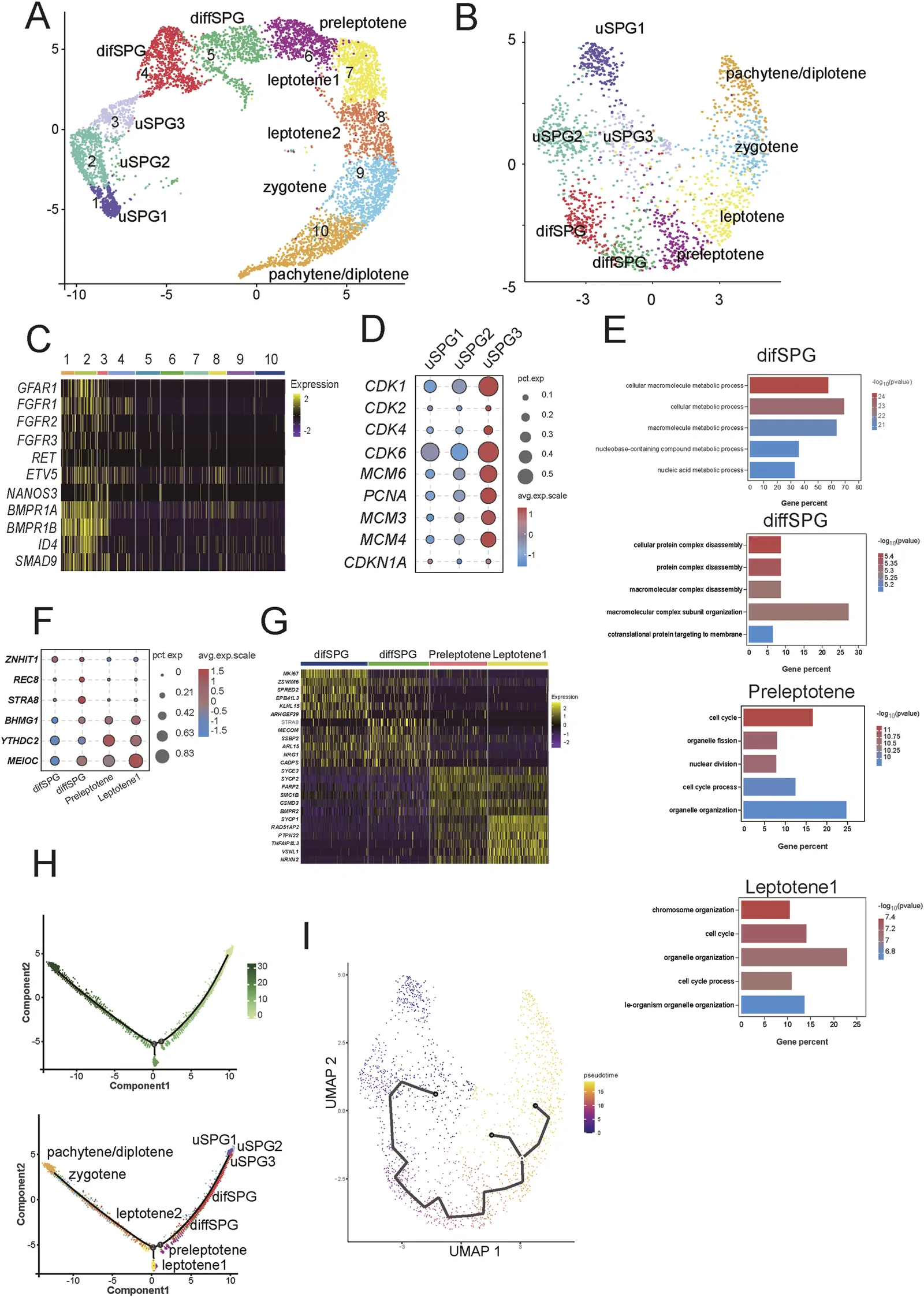
Subclustering of germ cells reveals novel spermatogonial subtypes and molecular features of meiotic entry. **(A)** Subclustering of germ cells from snRNA-seq of pubertal testis identifying 10 sequential stages: uSPG1-3, difSPG, diffSPG, preleptotene, leptotene1/2, zygotene, and pachytene/diplotene. **(B)** Corresponding subclustering based on snATAC-seq of pubertal testis. **(C)** Heatmap showing expression of self-renewal (GDNF/FGF/BMP pathway) genes across germ cell subclusters. **(D)** Cell-cycle activity inferred from expression of proliferation markers: uSPG3 is actively dividing, while uSPG1 and uSPG2 are quiescent. **(E)** GO enrichment for difSPG, diffSPG, preleptotene and leptotene1. **(F)** Expression of meiotic initiation regulators along the pseudotime trajectory. **(G)** Selected highly expressed genes in difSPG, diffSPG and preleptotene/leptotene cells. **(H)** Pseudotime trajectory (upper) and distribution of individual germ cell subclusters along the trajectory (down) based on pubertal snRNA-seq data. **(I)** Pseudotime trajectory of germ cells based on pubertal snATAC-seq data.

Based on snRNA-seq, germ cells in pubertal monkeys can be classified into 10 clusters including three uSPGs, one differentiating spermatogonia (difSPG), one differentiated spermatogonia (diffSPG), one preleptotene, two leptotenes, one zygotene, and one pachytene and diplotene stages (Figure 3A; Supplementary Figure S3A). Interestingly, the snRNA-seq data reveals a sequential expression pattern of genes associated with synaptonemal complex assembly. The genes *SYCP3*, involved in assembling lateral elements, and *SYCE1*, *SYCE3*, involved in assembling central elements, are expressed initially. Subsequently, the genes *SYCP2*, involved in assembling lateral elements, and *SYCE2*, *TEX12*, involved in assembling central elements, are expressed. Finally, the gene *SYCP1* involved in assembling transverse filaments is expressed. Consistent with these observations, cell type annotation and gene expression patterns inferred from snATAC-seq analysis were generally congruent with those obtained from scRNA-seq (Figure 3B, Supplementary Figure S3B).

The gene markers for germ cell identified in the scRNA-seq (Lau et al., 2020; Shami et al., 2020) exhibited predominantly concordant expression patterns with those observed in our snRNA-seq (Supplementary Figures S3C,S3D). However, a small subset of genes demonstrated distinct expression patterns. For instance, *NANOS3* is expressed in differentiating SPG identified in scRNA-seq by Lau et al., 2020, whereas in undifferentiated SPG identified in our snRNA-seq. Genes such as *ID4* and *UCHL1* showed a transient pattern of high expression followed by a decrease in our snRNA-seq, while maintaining sustained high expression levels in scRNA-seq by Lau et al., 2020 (Supplementary Figure S3C). Moreover, marker genes for undifferentiated SPG identified in scRNA-seq by Shami et al., 2020, including *MORC1*, *MSL3*, *TCF3*, *FMR1*, and *MAGEB2*, exhibited relatively elevated expression levels in undifferentiated SPG and SC based on our snRNA-seq (Supplementary Figure S3D).

### 2.4 Gene expression profiles in undifferentiated spermatogonia maintenance and self-renewal

Maintaining the self-renewal of undifferentiated spermatogonia particularly SSC is crucial for ensuring proper development and function of the reproductive system. Several pathways associated with SSC self-renewal, including the GDNF/FGF/RAS-MAPK pathway (*GFRA1*, *FGFR1*, *FGFR2*, *FGFR3*, *RET*, *ETV5*, *NANOS3*) and BMP pathway (*BMPR1A*, *BMPR1B*, *SMAD9*, *ID4*) were significantly enriched in all three types of undifferentiated SPG (Figure 3C). This reaffirms that these three subtypes indeed represent subpopulations within the germ cells with self-renewal capabilities. However, the roles of these pathways appear to differ. For instance, the GDNF pathway is highly specific to undifferentiated SPG cells, while FGFR functions in both undifferentiated and differentiating states.

Utilizing the expression profiles of cell cycle regulatory genes and functional enrichment analyses, it is plausible to deduce the cell types associated with the three subclasses of uSPGs. The expression profiles of cell cycle-associated genes reveal that uSPG3 is in an actively dividing state, as evidenced by elevated expression of *CDK1, CDK2, CDK4, CDK6, MCM6, PCNA, MCM3, MCM4,* and *CDKN1A* (Figure 3D). Among these, *CDK1* and *CDK2* are essential for G2/M and S phase progression, respectively. *CDK4* and *CDK6* promote G1/S transition. *MCM3, MCM4*, and *MCM6* are core replicative helicase components and *PCNA* functions as a DNA polymerase processivity factor. The proliferative uSPG3 potentially corresponds to the Ap subpopulation (A pale spermatogonia) in the classification of primate SSCs. The Ap cells undergo active mitosis, with one daughter cell retaining stem cell characteristics while the other differentiates into a progenitor cell (Hermann et al., 2010). The uSPG3 exhibits significant enrichment in signaling pathways governing nucleocytoplasmic transport, spliceosome and RNA degradation, which are associated with a self-renewal state (Hasegawa et al., 2013; Takashima et al., 2015) (Supplementary Figure S4A). In contrast, uSPG2 is quiescent with low expression of cell cycle genes (Figure 3D) and exhibits significant enrichment in signaling pathways governing the pluripotency of stem cells (Supplementary Figure S4A), suggestive of an Ad cell type (A dark spermatogonia). Similarly, uSPG1 is also quiescent (Figure 3D). Its transcriptomic profile and its position in the pseudo-time trajectory suggest a differentiation status that may be relatively early compared to the more classic type A SSCs (uSPG2 and uSPG3) (Figures 3A,B,H,I). This raises the possibility that it could represent a novel, quiescent subset of undifferentiated spermatogonia. Interestingly, we found that uSPG1 is enriched in the signaling pathways related to the nervous system with high expression of genes associated with neural synaptic function including *GRID2, NLGN1, LRRTM4, GRM1, GABRB1* and *GRIN2B* (Supplementary Figure S4C). We also investigated whether uSPG1 shares similarities with prespermatogonia at embryonic or neonatal stages. We examined the expression of a set of well-established marker genes for gonocytes, and showed low to undetectable expression of *POU5F1*, *NANOG*, *TFAP2C*, *DMRT1*, and *KIT* (Supplementary Figure S4D). Based on above, although our data point to a potentially distinct uSPG1 subtype, its precise characteristics and functions remain to be fully elucidated.

The identification of marker genes specific to the three subtypes of uSPGs could provide valuable insights into the molecular signatures defining each subtype (Supplementary Figure S4B). Notably, some of these genes are implicated in various signaling pathways, such as *PTCHD4* modulating the Hedgehog pathway and *FAM150B* participating in the ALK pathway. The intricate regulation of these signaling cascades is likely to exert a pivotal influence on shaping the functional characteristics and biological behaviors of SSCs, underscoring their significance in spermatogenesis and reproductive biology.

### 2.5 Gene expression profile of cell differentiation in meiosis

The transition from the mitotic phase to the meiotic phase in germ cells is a hallmark of the spermatogenesis process. To better understand the molecular mechanisms underlying cell differentiation in this process, we focused on 4 cell subtypes: difSPG, diffSPG, Preleptotene and Leptotene1.

Gene Ontology (GO) enrichment analysis revealed that difSPG exhibited enrichment in metabolic processes, while diffSPG showed enrichment in intricate disassembly and reassembly processes (Figure 3E). Preleptotene displayed enrichment in cell cycle, nuclear division, and organelle fission processes, whereas Leptotene1 demonstrated enrichment in chromosome organization, organelle biogenesis, and cell cycle processes (Figure 3E).

The chromatin remodeler *ZNHIT1*, a key player in meiotic initiation, manifested its expression primarily in difSPG (Figure 3F). Subsequent to this, the chromatin remodeler *REC8* and the core meiotic transcription factor *STRA8* were expressed in diffSPG (Figure 3F). Following this cascade, the *STRA8* targeted genes *BHMG1* (also known as *MEIOSIN*), *YTHDC2*, and *MEIOC* were expressed in Preleptotene (Figure 3F). These observations suggest that difSPG and diffSPG are primed for the onset of meiosis, with the meiotic program already underway in the diffSPG-Preleptotene transition. However, aside from *STRA8*, the identification of genes uniquely highly expressed in diffSPG posed challenges. Genes enriched in diffSPG encompassed *MECOM*, *SSBP2*, *APL15*, *NRG1*, and *CADPS*, albeit being transcriptionally active in difSPG (Figure 3G).

Transcriptomic pseudo-time analysis and ATAC pseudo-time analysis both yield expected unidirectional differentiation trajectories (Figures 3H,I). According to snRNA-seq, the process of differentiation appears to be continuous, with overlapping states observed between distinct cell types, except for a noticeable shift in cellular states between the preleptotene and leptotene. This indicated remarkable alterations in Preleptotene-Leptotene1 cells. The enriched genes in these 2 cell types include well-known essential genes in meiosis: *SYCP2*, *SYCE3*, *SMC1B*, *SYCP1*, and *RAD51AP2* (Figure 3G). Our data also revealed additional genes such as *FARP2*, *CSMD3*, *BMPR2*, TNFAIP8L3, *VSNL1*, *PTPN22*, and *NRXN2* (Figure 3G), which are likely crucial for the meiotic process in primate species. Among these, *BMPR2*, bone morphogenetic protein receptor type 2, has been implicated in spermatogenesis in several species. In mice, *Bmpr2* is among the most abundantly expressed BMP receptors in the postnatal testis (Ciller et al., 2016). Also, *bmpr2a* mutants phenocopied all phenotypes of the *amh* mutant in zebrafish and exhibit abnormal testis morphology, including spermatogonia accumulation and limited meiotic progression, directly linking BMPR2 signaling to meiosis (Zhang et al., 2020). TNFAIP8L3, TNF alpha-induced protein 8-like 3, has recently been identified as a novel meiosis candidate gene in human ovary. It positively regulates phosphoinositide 3-kinase activity, a pathway involved in cell growth, survival, and sensitivity to IGF-1 (McGlacken-Byrne et al., 2025). For the remaining genes *FARP2, CSMD3, VSNL1, PTPN22,* and *NRXN2*, previous studies provide limited evidence for a meiotic function, thus represent novel candidates whose functions in meiotic progression warrant further investigation.

### 2.6 Expression patterns of genes involved in spermatogenesis in sertoli cells

**FIGURE 4:**
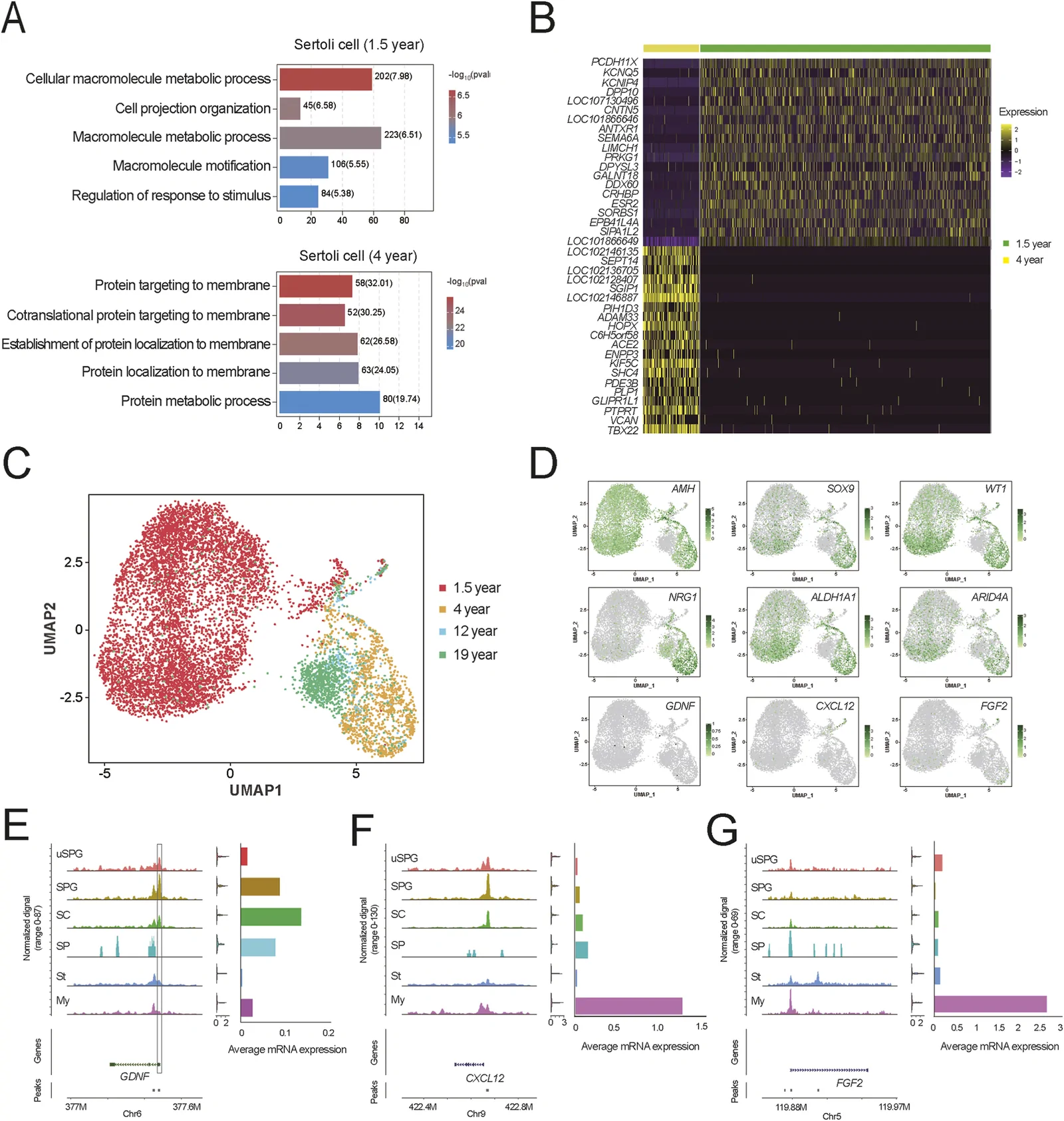
Sertoli cell heterogeneity and reevaluation of niche factor expression. **(A)** GO enrichment comparison between infant (1.5 years old) and pubertal (4 years old) Sertoli cells. **(B)** Representative highly expressed genes in infant (1.5 years old) and pubertal (4 years old) Sertoli cells. **(C)** UMAP of Sertoli cells subclustered, showing clear separation by age. **(D)** Expression of testis development-associated genes in Sertoli cells across ages. **(E–G)** SnRNA-seq (from all ages) and snATAC-seq (from pubertal testis only) tracks showing that *GDNF* **(E)** is not detected in Sertoli cells but is detectable in germ cells, while *CXCL12* **(F)** and *FGF2* **(G)** are primarily detected in myoid cells. For the pubertal stage, the chromatin accessibility data (snATAC-seq) showed no prominent open promoter regions in Sertoli cells for these three genes.

The analysis of single-nucleus transcriptomics revealed a higher abundance of Sertoli cells compared to single-cell transcriptomics. In combined analysis of all ages, Sertoli cells were classified into two distinct clusters, namely, cluster 11 and cluster 12 (Figures 1D,E). Cluster 11 primarily consisted of Sertoli cells from the infant testis, while cluster 12 mainly included those from the pubertal testis. Contrasting the differences between prepubertal and pubertal Sertoli cells enhances comprehension of changes in the somatic cell environment following the initiation of meiosis. Infant (1.5 years old) Sertoli cells exhibit enrichment in processes such as cellular macromolecule metabolic activities, cell projection organization, and macromolecule modification (Figure 4A). Genes significantly expressed in these cells include *PCDH11X*, *KCNQ5*, *KCNIP4*, *DPP10*, and *CNTN5* (Figure 4B). In contrast, Sertoli cells from pubertal (4 years old) monkey showed enrichment in processes related to protein targeting or localization to the membrane (Figure 4A), with highly expressed genes such as *SEPT14*, *SGIP1*, *PIH1D3*, *ADAM33*, and *HOPX* (Figure 4B). This corresponds to the functional role of Sertoli cells post sexual maturation, where mature Sertoli cells envelop germ cells, establishing intricate interactions that necessitate protein interactions on their cell membrane surface.

Upon subclustering Sertoli cells, they could still be distinctly divided into two major clusters. Sertoli cells in infancy (1.5 years old) clustered together, while Sertoli cells in puberty, adult and aged (4, 12 and 19 years old) formed another distinct group (Figure 4C). Genes associated with testis development, such as *AMH*, *SOX9*, and *WT1*, were predominantly expressed in infant and pubertal (1.5 and 4 years old) stages. Genes related to paracrine signaling in meiosis, including *NRG1*, *ALDH1A1*, and *ARID4A*, exhibited expression across all four samples (Figure 4D). The *CLU* gene, which functions in protecting germ cells from stress-induced apoptosis and facilitating spermatogenesis, was highly expressed in infant and pubertal Sertoli cells (1.5 and 4 years old) (Supplementary Figure S4E). Notably, genes associated with self-renewal of SSC, namely, *GDNF*, *CXCL12*, and *FGF2*, were not detected by snRNA-seq in Sertoli cells across all four ages (Figure 4D). Our snRNA-seq data, combined from all age groups, preliminarily indicated a trend of higher *GDNF* expression in germ cells compared to Sertoli cells (Figure 4E), whereas *CXCL12* and *FGF2* transcripts were predominantly detected in Myoid cells (Figures 4F,G). This expression pattern was corroborated at the chromatin level at the pubertal stage, as our snATAC-seq data revealed higher promoter accessibility for these genes in the same respective cell types (Figures 4E–G; Supplementary Figure S5A-C). These convergent transcriptomic and epigenomic data at the pubertal stage, together with transcriptomic data from other stages, point to a cell-type-specific regulatory program that may differ from the classical paradigm and emphasize the necessity for further validation and reassessment of gene expression related to SSC self-renewal in monkeys. Furthermore, genes previously thought to be expressed in Sertoli cells, such as *DRD4*, *PVRL2*, *ZFP36L1*, *MFGE8*, *LGALSL*, *CASKIN1*, *ESYT3*, and *DPYSL4*, are also not expressed in Sertoli cells (Supplementary Figure S4F).

### 2.7 Descriptive differences in testicular cell composition and gene expression among individual animals of different ages

**FIGURE 5:**
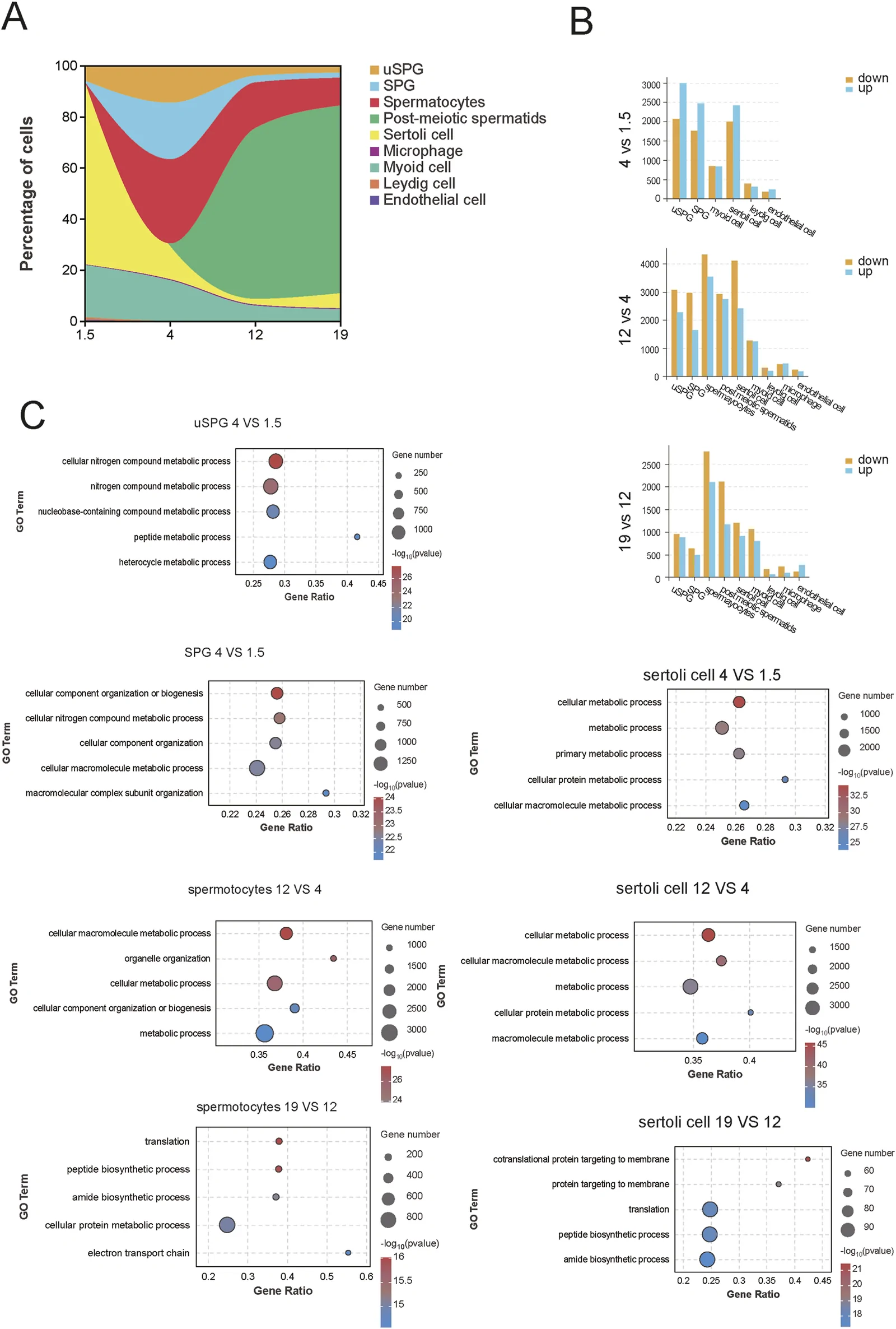
Descriptive differences in testicular cell composition and gene expression among individual animals of different ages. **(A)** Stacked river plot showing the percentage of each cell type captured by snRNA-seq among individuals of different ages. **(B)** Exploratory analysis of the number of DEGs (up-vs. downregulated) in each cell type among individuals of different ages. **(C)** Exploratory GO enrichment analysis of DEGs in selected cell types among individuals of different ages. One individual was included in each age group.

To descriptively compare the single-nucleus transcriptomes of testes from individual animals at different ages, we examined the cellular composition and gene expression profiles across the four samples. In the infant (1.5 years old) testis, somatic cells, particularly Sertoli cells and myoid cells, constituted the majority of the cellular population, accounting for over 90% of nuclei captured in snRNA-seq. The germ cells present at this stage were predominantly uSPG. In the pubertal (4 years old) testis, meiotic cells began to appear, and we observed that the combined percentage of uSPG, SPG, and spermatocytes reached over 60%. The cell composition in the adult (12 years old) testis suggested a more complete spermatogenesis process, encompassing all major types of germ cells and somatic cells. Post-meiotic cells appeared to account for over 50% in the adult testis, a proportion that seemed higher than that observed in the pubertal testis. The percentage of each cell type in the aged (19 years old) testis closely resembled that in the adult testis. When examining the sample-wise comparisons, we observed differences in cell type proportions across the four individual animals. For example, the proportion of pre-meiotic germ cells was highest in the pubertal sample, and lower in the infant, adult and aged samples. Post-meiotic germ cells accounted for a larger fraction of cell population in the adult and aged samples compared with the pubertal sample. The proportion of somatic cells was higher in the infant sample than in samples from the other three ages (Figure 5A).

When comparing gene expression between the infant (1.5 years old) and pubertal (4 years old) testis, we observed a tendency for more genes to be upregulated than downregulated in uSPG of pubertal testis (Figure 5B). These differentially expressed genes are predominantly enriched in processes related to nitrogen compound metabolism, nucleobase-containing compound metabolism, peptide metabolism, and heterocycle metabolism (Figure 5C). Among the most prominently upregulated genes in uSPGs were *CST9L*, *CST9LP1*, *SDF2L1*, *EPPIN*, and *CETN1* (Supplementary Figure S6A). Similarly, in SPGs there is a prevalence of upregulated genes over downregulated ones, with enrichment mainly observed in cellular component organization or biogenesis, cellular nitrogen compound metabolism, and cellular component organization (Figure 5C). The genes showing the most pronounced increases in SPGs included *DPEP3*, *RAD51AP2*, *GALNT14*, *SYCP2L*, and *CST9L.* Notably, *STRA8* is also among the genes showing elevated expression identified by snATAC-seq at the puberty time point, along with previously identified genes such as *MECOM* and *PRSS21*, which are potentially associated with initiating meiosis (Supplementary Figure S6B).

In pubertal (4 years old), adult (12 years old) and aged (19 years old) testis, spermatocytes exhibit the highest number of genes showing expression changes (Figure 5B). The transition from puberty to adulthood is characterized by enrichment in processes such as cellular macromolecule metabolism, organelle organization, and cellular metabolic processes (Figure 5C). During the aging process, there is a notable enrichment in processes related to translation, peptide biosynthesis, and amide biosynthesis (Figure 5C).

Among these stages, Sertoli cells exhibit the most numerous changes in gene expression among somatic cells (Figure 5B). The expression alterations from infancy to puberty and from puberty to adulthood in Sertoli cells primarily involve processes related to metabolic processes (Figure 5C) In the process of aging, difference is primarily enriched in protein targeting to the membrane (Figure 5C).

Nonetheless, as each age group was represented by a single animal, formal statistical testing for age-related effects is precluded. The findings presented here are descriptive and exploratory in nature. Future studies with larger cohorts will be essential to evaluate the developmental dynamics across ages in non-human primate.

## 3 Discussion

In this study, we aimed to gain novel insights into gene expression in monkey testis during spermatogenesis using single-nucleus-based analysis. Specifically, the study focused on the gene expression changes involved in maintaining self-renewal of spermatogonial stem cells and the transition of cells from mitosis to meiosis. Through single-nucleus level analysis, a greater number of cells was retained, providing a comprehensive understanding of gene expression patterns during cell differentiation, as well as the transition processes between different cellular subtypes.

By maintaining the self-renewal potential of SSCs, the testis ensures an adequate reservoir of stem cells to sustain continuous sperm production. The snRNA-seq and snATAC-seq data offer a novel perspective that aids in understanding the molecular mechanisms underlying undifferentiated spermatogonia self renewal. The identified pathways linked to self-renewal exhibit significant enrichment within SSCs, suggesting a pivotal role for these pathways in governing SSC function. However, comparison of the expression profiles of relevant genes indicates differential roles of these pathways. For instance, the GDNF/RET signaling pathway-related gene GFRA1 exhibits a high degree of specificity in uSPGs, suggesting that GFRA1 plays a unique role exclusively in uSPGs. GFRA1, acting as a co-receptor bound to the ligand GDNF, activates the RET receptor tyrosine kinase, thereby mediating downstream signal transduction (Takashima et al., 2015). This signaling pathway plays a crucial role in the proliferation and maintenance of SSCs (Jijiwa et al., 2008; Meng et al., 2000; Naughton et al., 2006). However, FGFRs are expressed in both undifferentiated and differentiating spermatogonia, suggesting that the role of FGF/FGFR extends beyond maintaining SSC self-renewal. Both *in vitro* and vivo studies have demonstrated that FGFs can maintain the undifferentiated state and promote proliferation of SSC (Takashima et al., 2015; Kanatsu-Shinohara et al., 2003; Kubota et al., 2004). Our research findings further support the multifunctionality of FGF/FGFR in germ cell development. These findings underscore the intricate regulatory networks and molecular mechanisms involved in the differentiation and self-renewal of undifferentiated spermatogonia.

Based on the intensity of nuclear staining, undifferentiated SPG in monkey and human can be classified into two types, Ad and Ap. The Ad SPG are mitotically quiescent and serve as reserve stem cells, while Ap SPG are actively undergoing mitosis, replenishing themselves, and generating the initial generation of differentiated SPG (Hermann et al., 2010). Based on scRNA-seq data, Shami et al., 2020 (Shami et al., 2020) identified two subpopulations of undifferentiated SPG, which were not found to correspond to the Ap and Ad cell states in human. In contrast, the results of this study closely align with cell types identified based on nuclear staining. Nuclear transcriptome data identified three distinct uSPG cell types, with two potentially corresponding to Ap SPG, and Ad SPG. The third cell subtype uSPG1 likely represents a transcriptionally distinct subset of undifferentiated spermatogonia, possibly positioned early in the differentiation hierarchy based on its snRNA-seq and snATAC-seq profile. Unfortunately, our dataset lacks sufficient evidence to conclusively characterize the molecular identity or functional properties of uSPG1. Additionally, we identified that uSPG1 is enriched in the signaling pathways related to the nervous system and expressed several genes classically associated with synaptic function. The association between spermatogenesis and the nervous system is not a novel concept. Genes that play roles in the nervous system also contribute to spermatogenesis, such as *GDNF* (Meng et al., 2000; Kawai and Takahashi, 2020). While this observation is intriguing and echoes the known molecular parallels between the brain and testis (Matos et al., 2021), we interpret this finding with caution. Such pathway enrichment could be a consequence of the pleiotropic functions of these genes and the gene annotation databases used, rather than direct evidence of neural-like features. Further investigation is necessary to understand the functional significance of this expression pattern.

The entry of germ cells into meiosis represents a critical cellular fate transition and is a focal point in the process of spermatogenesis. Retinoic acid and its target *STRA8* have been shown to be necessary, while they are not sufficient to induce the initiation of mammalian germ cell meiosis (Pfaltzgraff et al., 2024). There is still a need to investigate the molecular mechanisms that initiate meiosis. Single-nuclear transcriptomic data revealed genes such as *ZNHIT1*, *REC8*, *STRA8*, *BHMG1* (*MEIOSIN*), *YTHDC2*, and *MEIOC*, which play pivotal roles in the initiation of meiosis (Pfaltzgraff et al., 2024; Anderson et al., 2008; Bailey and Fuller, 2024; Bannister et al., 2004; Ishiguro et al., 2020; Sun et al., 2022), are significantly expressed in the corresponding cell clusters. Additionally, diffSPG is identified as a key cell population during the initiation of meiosis. The top 5 highly expressed genes in this population are *MECOM*, *SSBP2*, *ARL15*, *NRG1*, and *CADPS*. *MECOM* has been reported to encode a transcriptional factor involved in hematopoiesis, apoptosis, development, and proliferation (Zhang et al., 2011). *MECOM* is also significantly enriched in SPG transitioning from the infancy to puberty, suggesting their potential involvement in initiating meiosis. *SSBP2* is a subunit of the single-stranded DNA binding complex, involved in maintaining hematopoietic stem cells and stress response (Li et al., 2014). The specific roles of *SSBP2* in spermatogenesis have not been reported, highlighting the need for further research in this area.

During meiosis, the assembly of the synaptonemal complex (SC) is a critical event that occurs between paired homologous chromosomes. It is essential for ensuring the correct progression of homologous recombination. The SC is a protein complex resembling a zipper structure, consisting of two parallel lateral elements (LE), a central element (CE) located between the lateral elements, and transverse filaments (TF) (Costa and Cooke, 2007). During the leptotene stage, SYCP2 and SYCP3 proteins are recruited to the chromosome axis. In the zygotene stage, homologous chromosomes pair up, forming the central axis of the synaptonemal complex (SC) between the paired lateral elements, completing the SC assembly (Costa and Cooke, 2007). According to snRNA sequencing data, the assembly of SC components follows a specific sequential expression pattern. Initially, genes involved in assembling the lateral elements, such as *SYCP3*, and those involved in assembling the central elements, such as *SYCE1* and *SYCE3*, are expressed. Subsequently, genes involved in assembling the lateral elements, like *SYCP2*, and those involved in assembling the central elements, such as *SYCE2* and *TEX12*, are expressed. Finally, the gene responsible for assembling the transverse filaments, *SYCP1*, is expressed. The sequential gene expression pattern indicates precise temporal regulation during meiosis, facilitating the timely appearance of each component to ensure accurate assembly.

Based on the snRNA-seq, we obtained a larger number of Sertoli cells, which is markedly distinct from previous studies. Shami et al., 2020 (Shami et al., 2020) collected testes from five monkeys but failed to identify Sertoli cells. Lau et al., 2020 (Lau et al., 2020) collected testes from four monkeys, while the identified Sertoli cells represent the least abundant cell type among somatic cells. Sertoli cells primarily provide structural support for germ cells, typically in proportion to the number of germ cells. In the human testis, their quantity is approximately 1/1.5–1/2 of germ cells (Johnson et al., 2008). Our study identified a significant population of Sertoli cells, aligning well with previous knowledge. Furthermore, we have gained novel insights into the functions of Sertoli cells. It has been traditionally believed that Sertoli cells play crucial roles in SSC self-renewal and differentiation (Meng et al., 2000; De Rooij, 2017). Notably, the expression of *GDNF*, *FGF2* and *CXCL12,* which plays a crucial role in SSC self-renewal and previously thought to be expressed in Sertoli cells, was not detected in Sertoli cells by our snRNA-seq and snATAC-seq datasets. We re-examined the published scRNA-seq data from Lau et al. (2020) (5), focusing on whether the expression patterns of *GDNF, FGF2*, and *CXCL12* across cell types within this dataset is consistent with our observations. In Lau et al. (2020), neither *GDNF* nor *FGF2* was reported as differentially upregulated genes in any cell type, indicating low or non-differential expression throughout. *CXCL12* was identified as upregulated in myoid cells, which is consistent with our finding, and importantly, was low or non-differential expressed in Sertoli cells. Thus, the expression patterns in Lau et al. (2020) are generally concordant with our observation. Nervertheless, we acknowledge that these findings in *macaca fascicularis* are preliminary and need further definitive experimental validation. If this observation is corroborated by future studies, it would suggest a need to revise our long-standing understanding of Sertoli cell function in spermatogenesis of non-human primate. We also recognize that our finding contrasts with studies conducted in other species, for example, in Johnston et al. (2011), they demonstrate stage-specific *Gdnf* expression by Sertoli cells in rat (Johnston et al., 2011). These discrepancies might be explained by differences in methodology and species. Johnston et al. (2011) localized GDNF protein within intact seminiferous tubules using immunohistochemistry and confocal microscopy. Although powerful for spatial visualization, these methods are inherently limited in their resolution when distinguishing individual cell types within the tightly packed epithelium, particularly in the absence of co-staining with a Sertoli cell marker (e.g., AMH or SOX9). Johnston et al. (2011) also used RT-PCR to analyze *Gdnf* mRNA but relied on manually isolated Sertoli cells, a method inherently prone to contamination by adjacent germ cells or other somatic cells. Moreover, GDNF is an excretory protein, the localization of the protein and mRNA does not indicate the cell type in which transcription occurs. Our snRNA-seq and snATAC-seq approaches provide a single-nuclear level readout of transcriptional activity and chromatin accessibility, which can help inform the mapping of GDNF transcripts to their cells of origin. Additionally, rodent and primate testes differ in their spermatogonial stem cell heterogeneity and signaling networks. It is therefore plausible that the cellular source of *GDNF* has diverged between these species. It is also worth mentioning that misconceptions can arise due to cellular behaviors of Sertoli cells. Since Sertoli cells often stretch their cytoplasm to communicate directly with developing germ cells (Oatley and Brinster, 2012) and engulf germ cell remnants (Yefimova et al., 2018), signals attributed to Sertoli cells may in fact derive from closely associated or internalized germ cells, potentially confounding interpretations of Sertoli cell gene expression. For example, *PRM2*, previously thought to be expressed in Sertoli cells, was found to be an illusion caused by Sertoli cells engulfing germ cells (Wykes et al., 1995). While Sertoli cells are widely regarded as a primary source of niche factors in mammals, some evidence suggest that the cellular sources of *GDNF* may be more complex than previously appreciated. For instance, studies in mice have demonstrated that peritubular myoid cells also produce *GDNF* and that testosterone-induced *GDNF* expression in these cells contributes to spermatogonial stem cell maintenance (Chen et al., 2014). Conditional knockout of the *Gdnf* gene specifically in peritubular myoid cells resulted in severe depletion of undifferentiated spermatogonia and male infertility (Chen et al., 2016). In addition to *GDNF*, genes such as *DRD4, PVRL2, ZFP36L1, MFGE8, LGALSL, CASKIN1, ESYT3, DPYSL4*, which were previously thought to be expressed by Sertoli cells, were also not found to be expressed by Sertoli cells in our snRNA-seq data. These results also require further experimental verification.

Single-nucleus RNA sequencing technology plays a crucial role in biological studies and has been widely applied in various research fields. Firstly, snRNA-seq is highly similar to scRNA-seq, enabling effective identification of cell populations (Bakken et al., 2018; Slyper et al., 2020). Secondly, the use of snRNA-seq technology can effectively overcome issues of cell damage during digestion, thereby capturing a more diverse range of cell types. Our study demonstrates that snRNA-seq successfully captured Sertoli cells that were not identified in previous scRNA-seq studies (Shami et al., 2020), providing potential for in-depth research on it. In terms of sample processing and preservation, the snRNA-seq method also demonstrates convenience. Nonetheless, we have to point out that snRNA-seq has inherent technical constraints that could influence observations. SnRNA-seq captures only nuclear RNA, which represents a fraction of the total cellular transcriptome and may be biased towards nascent or unspliced transcripts, while cytoplasmic transcripts, especially those with rapid turnover or involved in local translation are entirely missed. This leads to a systematic underestimation of the expression of many genes, particularly those with a predominantly cytoplasmic localization or those whose function relies on immediate translation. Simultaneous application of scRNA-seq and snRNA-seq may help to comprehensively understand the fine-tuning mechanisms of gene expression. For example, by comparing snRNA-seq and scRNA-seq data, we revealed differences between nuclear transcriptomes and cell transcriptomes. Specifically, for genes such as *NR6A1*, *MORC1* and *BRDT* that were differently expressed in snRNA-seq and scRNA-seq datasets, the observed differences reflect distinct transcriptional regulatory mechanisms underlying the expression of spermatogenesis-related genes. Some of these genes require continuous transcription to maintain mRNA stability, whereas others can maintain mRNA stability without the need for continuous transcription activity. Another limitation of the current study is that each developmental stage was represented by a single animal. This precludes formal statistical testing for age-related effects and makes it difficult to exclude the possibility that individual-specific or batch-related variations might have contributed to the observed transcriptomic differences across age groups. Future studies with larger cohorts will be essential to evaluate the spermatogenesis process in non-human primate.

In conclusion, our research advances the comprehension of gene expressions that regulate spermatogenesis in monkeys through single-nucleus-based analysis. The results of this study not only identified key genes and cell populations involved in the undifferentiated spermatogonia self-renewal and the initiation of meiosis, but also underscore the importance of investigating the functions of Sertoli cells in male reproductive health. Delving deeper into the regulatory mechanisms uncovered in this study could potentially pave the way for novel therapeutic approaches aimed at addressing male infertility and reproductive disorders.

## 4 Materials and methods

### 4.1 Testicular tissue samples

Fixed and snap-frozen testicular tissues were obtained from four male cynomolgus macaques (*Macaca fascicularis*) comprised one infancy (1.5 years old), one puberty (4 years old), one adult (12 years old), and one aged (19 years old) individuals. Testicular tissues have been fixed in 4% paraformaldehyde (PFA) in phosphate-buffered saline (PBS, pH 7.4) at 4 °C for 24 h and then embedded in paraffin for histological analysis, or snap-frozen in liquid nitrogen and stored at −80 °C for single-nucleus isolation. The snRNA-seq dataset was generated from all four individuals, while the snATAC-seq dataset was generated exclusively from the pubertal (4 years old) individual. All experimental procedures were approved by the Institutional Animal Care and Use Committee of Yunnan University (Approval No. YNU20210110).

### 4.2 Histological examination (H&E staining)

Paraffin-embedded testicular blocks were sectioned at 5 µm thickness using a rotary microtome (Leica CM3050S). Sections were mounted on SuperFrost Plus slides and dried overnight at 37 °C. After deparaffinization in xylene and rehydration through a descending ethanol series, slides were stained with hematoxylin for 5 min, differentiated in 0.3% acid alcohol, and counterstained with eosin for 2 min. The sections were then dehydrated, cleared, and mounted with neutral balsam. Images were captured with a light microscope (ZEISS Imager M2) equipped with a digital camera.

### 4.3 Single-nucleus RNA sequencing (snRNA-seq)

One individual was included in each age group. Frozen testicular tissues (∼50 mg per sample) from all four infant, pubertal, adult and aged individuals were homogenized in ice-cold lysis buffer (0.1% Triton X-100, 0.1% Tween-20, 0.01% BSA, 0.4 U/μL RNase inhibitor in PBS) using a Dounce homogenizer. The homogenate was filtered through a 40 μm cell strainer and centrifuged at 500 *g* for 5 min at 4 °C. The nuclear pellet was resuspended in washing buffer (1% BSA in PBS) and the concentration was adjusted to approximately 1,000 nuclei/µL. Nuclei integrity and purity were assessed by trypan blue staining and microscopic examination. Single-nucleus suspensions were loaded onto a Chromium Controller (10x Genomics) to generate Gel Bead-in-Emulsions (GEMs) targeting 10,000 nuclei per sample. Libraries were constructed using the Chromium Next GEM Single Cell 3′Reagent Kit v3.1 according to the manufacturer’s protocol. Final libraries were quantified by Qubit 4.0 fluorometer and Agilent 2,100 Bioanalyzer, and sequenced on an Illumina NovaSeq 6,000 platform with a paired-end 150 bp (PE150) configuration.

### 4.4 Single-nucleus ATAC sequencing (snATAC-seq)

Frozen testicular tissue from one pubertal (4 years old) individual was subjected to nuclei isolation as described above for snRNA-seq, with the following modifications. After the nuclei pellet was obtained, it was resuspended in 1× Diluted Nuclei Buffer (10x Genomics). The nuclei were counted, and ∼50,000 intact nuclei were used for transposition. Transposition was performed using the Chromium Next GEM Single Cell ATAC Kit (10x Genomics) according to the manufacturer’s protocol: nuclei were permeabilized, incubated with Tn5 transposase (37 °C, 30 min), and then washed. Approximately 10,000 transposed nuclei were loaded onto a Chromium Controller for GEM generation and library preparation. Libraries were sequenced on an Illumina NovaSeq 6,000 platform with paired-end 50 bp (PE50) reads.

### 4.5 Data processing and bioinformatics analysis

#### 4.5.1 snRNA-seq data processing

Raw base call (BCL) files were converted to FASTQ format using Cell Ranger (v6.0, 10x Genomics). Reads were aligned to the *Macaca fascicularis* reference genome (Macaca_fascicularis_6.0) using the cellranger count pipeline with default parameters. Gene-barcode matrices were generated. To identify and eliminate potential multiplets, we used the DoubletFinder package (v2.0.3). Briefly, for each sample, the paramSweep function was first used to determine the optimal pK parameter for neighborhood analysis. Subsequently, the expected multiplet rate was calculated based on the relationship between the effective number of recovered nuclei and the multiplet rate provided by the 10x Genomics. This expected rate was used as the filtering threshold. The doubletFinder function was then applied to calculate the pANN (probability of being a nearest neighbor for a multiplet) value for each GEM. GEMs with a pANN value exceeding the sample-specific expected multiplet rate were classified as multiplets and removed from downstream analyses. Low-quality cells with fewer than 340 or more than 7,100 detected genes, as well as those with >10% mitochondrial reads, were filtered out using Seurat (v4.3). After quality control, 43,902 high-quality nuclei were retained for downstream analysis. Data normalization, scaling, and identification of highly variable genes were performed using Seurat’s NormalizeData, ScaleData, and FindVariableFeatures functions. Principal component analysis (PCA) was run on the variable genes, and the top 30 principal components (PCs) were used for Uniform Manifold Approximation and Projection (UMAP) dimensionality reduction and graph-based clustering (FindNeighbors, FindClusters, resolution = 0.6). To minimize potential batch effects arising from library preparation across different age groups, we applied Harmony (v0.1.1) to integrate the four samples prior to clustering and visualization. Specifically, we used the RunHarmony function with the “batch” parameter set to sample origin, using previously identified PCs as input. This step projects cells into a shared embedding where clustering is driven by cell-type identity rather than dataset-specific technical variation. The Harmony-corrected embeddings were then used for UMAP dimensionality reduction and graph-based clustering. For graph-based clustering, we constructed a shared-nearest neighbor (SNN) graph using the FindNeighbors function with the Harmony-corrected PCs, where edges between cells were weighted based on the Jaccard similarity of their local neighborhoods. Clusters were then identified using the Louvain algorithm via FindClusters with a resolution parameter of 0.6. For visualization, we generated both UMAP and t-distributed stochastic neighbor embedding (t-SNE) projections using the same corrected PC space. To identify upregulated genes specific to each subcluster, we additionally applied a likelihood-ratio test with the following criteria (Edenfield et al., 2025): P-value ≤ 0.01 (Griswold, 2018); log2 (fold change) ≥ 0.36; and (Chen et al., 2018) the percentage of cells expressing the gene within the target cluster > 25%. Differential expression analysis was conducted with the FindAllMarkers function using the Wilcoxon rank-sum test (|log2 fold change| > 0.25, adjusted p-value < 0.05). Cell types were annotated based on canonical marker genes.

#### 4.5.2 snATAC-seq data processing

Raw sequencing data were processed using Cell Ranger ATAC v2.0 (10x Genomics) with default parameters against the *Macaca fascicularis* reference genome (Macaca_fascicularis_6.0). The resulting fragment files were used for downstream analysis with Signac v1.9 and Seurat v4.3. Cells with low quality were filtered out using the following criteria: nucleosome signal > 4, TSS enrichment score < 2, and total fragments < 1,000. After filtering, 5,103 high-quality nuclei were retained. Latent semantic indexing (LSI) was performed on the peak-by-cell matrix, and the top 30 LSI components were used for UMAP dimensionality reduction and graph-based clustering. Chromatin accessibility peaks were called using MACS2 integrated in Signac.

#### 4.5.3 Integration of snRNA-seq and snATAC-seq

A gene activity matrix for snATAC-seq was generated using Signac’s GeneActivity function. Seurat’s label transfer approach was applied to project snATAC-seq cells onto the snRNA-seq UMAP embedding: anchors were identified between the snRNA-seq gene expression matrix and the snATAC-seq gene activity matrix using FindTransferAnchors, and snATAC-seq cells were mapped via TransferData. For comparative analyses, we further performed co-embedding using Harmony with canonical correlation analysis (CCA) components from both modalities.

#### 4.5.4 Gene ontology and pathway enrichment

GO enrichment analysis was performed using clusterProfiler (v4.6) with a threshold of adjusted p-value < 0.05. All protein-coding genes detected in our snRNA-seq dataset were used as the background gene set for enrichment calculations. Specifically, differentially expressed genes for each cluster were mapped to GO terms in the GO database (http://www.geneontology.org/), and significantly enriched terms were identified using a hypergeometric test with false discovery rate (FDR) correction applied. Terms with FDR ≤ 0.05 were considered significantly enriched. For KEGG pathway enrichment analysis (https://www.kegg.jp/), the same background set and FDR threshold (≤0.05) were applied to identify significantly enriched metabolic and signaling pathways. Visualization was conducted using ggplot2 and ComplexHeatmap.

#### 4.5.5 Pseudotime trajectory analysis

Pseudotime trajectories for germ cell differentiation were reconstructed using Monocle3 (v1.3) on snRNA-seq data and on snATAC-seq data after gene activity score calculation.

#### 4.5.6 Motif enrichment analysis

Transcription factor motif enrichment in snATAC-seq peaks was assessed using chromVAR (v1.20) with the JASPAR2022 motif database.

## Data Availability

The datasets presented in this study can be found in online repositories: Genome Sequence Archive (Genomics, Proteomics & Bioinformatics 2025) in National Genomics Data Center (Nucleic Acids Res 2025), China National Center for Bioinformation / Beijing Institute of Genomics, Chinese Academy of Sciences (GSA: CRA045074) that are publicly accessible at https://ngdc.cncb.ac.cn/gsa.

## Supplementary material

SUPPLEMENTARY FIGURE S1 Validation of cell-type annotations by snRNA-seq and comparison with published single-cell datasets. **(A)** GO enrichment of differentially expressed genes in each major cell type, confirming their expected biological processes. **(B,C)** Comparison of marker gene expression patterns between our snRNA-seq data and two published scRNA-seq datasets from Lau et al., 2020 **(B)** and Shami et al., 2020 **(C)**. Representative cell types and marker genes identified in published scRNA-seq datasets (Y axis) were mapped onto the clustering result of our snRNA-seq data (X-axis). Abbreviations: GC, germ cells; SPG, spermatogonia; SC, spermatocytes; RSs, round spermatids; ESs, elongated spermatids; sperm, spermatozoa; SoC, somatic cells; St, Sertoli cells; Ld, Leydig cells; My, myoid cells; Ed, endothelial cells; STid, spermatid.

SUPPLEMENTARY FIGURE S2 Chromatin accessibility and expression of marker genes across cell types in pubertal testis identified by snATAC-seq and snRNA-seq. **(A)** Dot plot showing the chromatic accessibility of marker genes across the six cell types identified by snATAC-seq. **(B)** Dot plot showing the expression of marker genes across the eight cell types identified by snRNA-seq.

SUPPLEMENTARY FIGURE S3 Detailed gene expression heatmaps for germ cell subclusters. **(A)** Dot plot showing the expression of stage-specific marker genes across the 10 germ cell subclusters identified by snRNA-seq of pubertal testis. Genes are ordered according to their known function in spermatogonial self-renewal, meiosis initiation, synaptonemal complex assembly and post-meiotic differentiation. **(B)** Dot plot showing of chromatic accessibility of marker genes across subclusters identified by snATAC-seq of pubertal testis. **(C–D)** Comparison of germ cell marker gene expression patterns between our snRNA seq data and two published scRNA seq datasets from Lau et al., 2020 **(C)** and Shami et al., 2020 **(D)**. Representative marker genes identified in published scRNA-seq datasets (Y axis) were mapped onto the clustering result of our snRNA-seq data (X-axis).

SUPPLEMENTARY FIGURE S4 Functional enrichment and gene expression in SSC uSPG subclusters and Sertoli cells. **(A)** KEGG pathway enrichment analysis for uSPG1-3 subclusters. **(B)** Heatmap of top marker genes distinguishing uSPG1, uSPG2, and uSPG3, which correspond to prespermatogonia (PreSPG), uSPG2 and uSPG3 correspond to Ad SPG, and Ap SPG in non-human primates, respectively. **(C)** Dot plot showing expression of genes related to nervous system in uSPG1-3 subclusters. **(D)** Dot plot showing expression of prespermatogonia markers genes in uSPG1-3 subclusters. **(E)** UMAP distribution of CLU expression across Sertoli cell clusters. **(F)** Violin plots showing that genes previously reported to be expressed in Sertoli cells (DRD4, PVRL2P2RV2, ZFP36L1, MFGE8, LGALSL, CASKIN1, ESYT3, DPYSL4) are not detected in Sertoli cells in our snRNA-seq data.

SUPPLEMENTARY FIGURE S5 Genome browser tracks showing chromatin accessibility at the GDNF, FGF2, and CXCL12 loci across testicular cell types. **(A–C)** Integrated Genomics Viewer (IGV) tracks displaying snATAC-seq signal coverage (normalized signal) and gene locations for GDNF **(A)**, FGF2 **(B)**, and CXCL12 **(C)** in the pubertal (4 years old) testis. Tracks are shown for six cell types: undifferentiated spermatogonia (uSPG), differentiating/differentiated spermatogonia (SPG), spermatocytes (SC), spermatids (SP), Sertoli cells (St), and myoid cells (My).

SUPPLEMENTARY FIGURE S6 Differentially expressed genes in uSPG and SPG from Infancy to Puberty. **(A)** Volcano plot showing differentially expressed genes in uSPG from Infancy (1.5 years old) to Puberty (4 years old). Top five genes with significant upregulation are highlighted and labeled. **(B)** Volcano plot showing differentially expressed genes in SPG from Infancy (1.5 years old) to Puberty (4 years old). Top eight genes with significant upregulation are highlighted and labeled.
